# Assessing and Strengthening African Universities' Capacity for Doctoral Programmes

**DOI:** 10.1371/journal.pmed.1001068

**Published:** 2011-09-13

**Authors:** Imelda Bates, Richard Phillips, Ruby Martin-Peprah, Gibson Kibiki, Oumar Gaye, Kamija Phiri, Harry Tagbor, Sue Purnell

**Affiliations:** 1 Disease Control Strategy Group, Liverpool School of Tropical Medicine, Liverpool, United Kingdom; 2 Kwame Nkrumah University of Science and Technology, Kumasi, Ghana; 3 Komfo Anokye Teaching Hospital, Kumasi, Ghana; 4 Kilimanjaro Clinical Research Institute, Kilimanjaro Christian Medical Centre, Moshi, Tanzania; 5 Department of Medical Parasitology, Dakar Université Cheikh Anta Diop, Dakar, Senegal; 6 College of Medicine, University of Malawi, Blantyre, Malawi

## Abstract

Imelda Bates and colleagues developed and validated an evidence-based tool for evaluating doctoral programmes in African universities.

**Summary Points**

- Universities can make a major contribution to good policy-making by generating nationally relevant evidence, but little is known about how to strategically support universities in poorer countries to train and nurture sufficient internationally competitive researchers.
- It is difficult for universities to develop a coherent strategy to identify and remedy deficiencies in their doctoral training programmes because there is currently no single process that can be used to evaluate all the components needed to make these programmes successful.
- We have developed an evidence-based process for evaluating doctoral programmes from multiple perspectives that comprises an interview guide and a list of corroborating documents and facilities; we refined and validated this process by testing it in five diverse African universities.
- The strategy and priority list that emerged from the evaluation process facilitated “buy-in” from internal and external agencies and enabled each university to lead the development, implementation, and monitoring of their own strategy for remedying doctoral programme deficiencies.

## Relationship between Indigenous Doctoral Programmes in Africa and Better Health Outcomes

The generation of local research, and the ability to innovate and to use research results, are essential for good policy making and ultimately for better health outcomes [1]. Research for policy should be led by a country's own scientists [2],[3], but very few universities in low-income African countries are able to “home grow” sufficient world class researchers [4]. Traditionally, doctoral students from low-income countries have been trained overseas, where they often learn skills they cannot use when they return home. Consequently there has been a recent shift in funding emphasis towards supporting students to remain in their home institution.

Implicit in this new approach is the need to strengthen African universities' capacity to deliver doctoral programmes, and to focus efforts not only on the training itself but also on creating an enabling environment for research, in particular, leadership, career development, infrastructure, and access to information [4]. Currently, the international academic community has insufficient understanding of the policies and processes required to develop research capacity in African universities, and this makes it difficult to target resources strategically towards priority capacity gaps [5].

## Lack of Methodologies to Evaluate Gaps in Universities' Policies and Processes for Doctoral Programmes

To enhance the capacity of African universities to run doctoral programmes, the Malaria Capacity Development Consortium (MCDC) [6] has funded 19 African researchers to undertake doctoral training in universities in Ghana, Malawi, Senegal, Tanzania, and Uganda. The doctoral programmes in each of the African universities were at different stages of maturity at the start of the programme, with the number of registered doctoral students varying from none to 147. The doctoral programme coordinators in each institution recognised that having a cohort of new doctoral students provided an opportunity for their institutions to strengthen their systems for doctoral programmes or, for those universities just starting doctoral programmes, to develop the necessary structures and processes. The African project coordinators therefore asked the MCDC secretariat for support in identifying ways in which their doctoral programmes could be improved. The project was advertised, and following a selection process, a contract to evaluate the African doctoral programmes was awarded to a team of researchers from the United Kingdom and Africa (IB, RP, RM-P, and SP).

A process was needed to identify gaps in the African institutions' existing doctoral programmes, so discussions between representatives from the universities and our research team defined the critieria that should be met by such a process (Box 1). However, published information about evaluating capacity development is very scarce [7], and a search of the literature failed to identify any single process that could be used to evaluate all the policies and processes needed to run successful doctoral programmes. The purpose of our study was therefore to develop and test an evidence-based process that could be used to evaluate all the components of doctoral programmes and to standardise the process so that it is transferable across universities.

**Box 1. Criteria to Be Fulfilled by the Evaluation Process**

The process should enable the university authorities to

- review all aspects of their doctoral programmes
- identify gaps in their capacity to manage these programmes
- develop strategies to remedy these gaps
- generate indicators that can be used to evaluate progress in filling these gaps

## Development of the Process for Evaluating Policies and Systems for Doctoral Programmes in Africa

**Figure 1:**
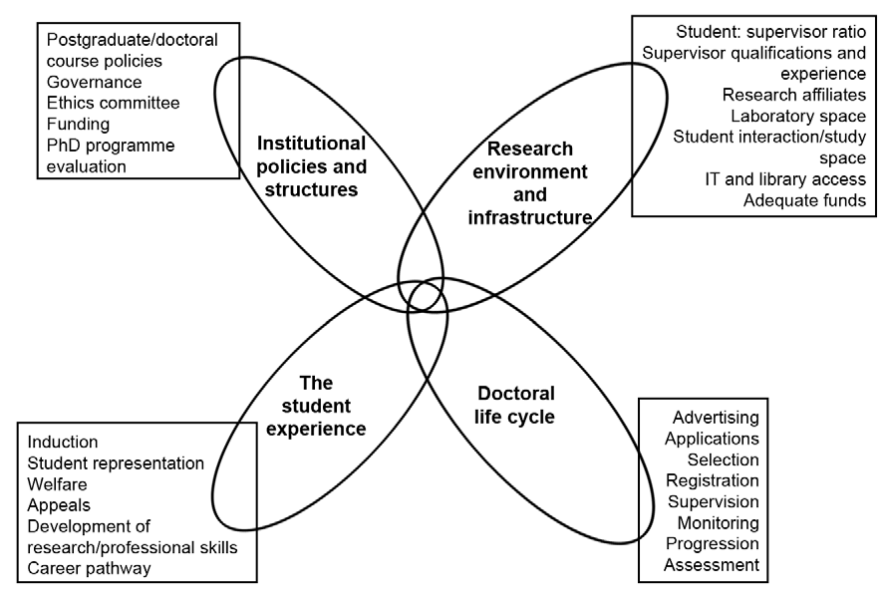
The four components of doctoral programmes, with examples of the constituents of the components.

To ensure that the final evaluation process was robust, it was derived from published evidence. We scanned published literature, Web sites and documents from universities, educational agencies, and regulatory bodies to identify and synthesize existing methods for evaluating any aspect of doctoral programmes (Box 2). Through this process we produced a list of all the policies, processes, and facilities needed to run doctoral programmes (summarised in Figure 1). We amalgamated all the information obtained into a draft evaluation process, which consisted of a list of stakeholders to be interviewed, an interview guide for each of the different cadres of stakeholders, a list of documents to be reviewed, and a list of facilities to be visited.

**Box 2. Examples of Sources of Information about Doctoral Policies and Processes**

- Code of practice from the UK Quality Assurance Agency [11] (used as a platform for incorporating other pieces of information)
- Institutional quality standards for the contents of doctoral programmes and research skills needed by doctoral students [12]–[14]
- Handbook and checklist for managing quality assurance in education programmes [15]
- Framework for conducting an assessment of institutional health research capacity [16]
- Personal development plan for African doctoral students [17]

## Testing and Finalising the Evaluation Process

The evaluation took place during site visits of 2–3 days to each of the five African universities that are partners in MCDC. These visits were preceded by pro-active engagement of key individuals in the universities who would facilitate the evaluations. The research team conducting the evaluation was independent of the MCDC managers, and the team's four members had expertise in health care delivery, research and doctoral supervision, academic and health care systems, and educational development. Interview bias was reduced by using different combinations of research team members to conduct the interviews.

Initially no assumptions were made about which questions could be answered by which interviewee, and all interviewees were asked every question. Interviewees were enthusiastic about being interviewed, but they were able to provide reliable information only about the aspects of doctoral programmes in which they were directly involved, and so there were many instances where inaccurate or incomplete information was provided. We were therefore meticulous about double-checking all the information we were given by asking the same question of more than one individual. Responses from the interviewees were corroborated by referring to institutional documents and directly observing facilities as appropriate. Any discrepancies were resolved through discussions between the researchers and the doctoral programme coordinators in each institution.

As the evaluations progressed we were able to match questions to specific types of interviewees, thereby improving the efficiency of the interviews. By honing the questions, removing duplications and adding new interviewees or observations to the process, as suggested by interviewees, we refined and streamlined the process and reduced the interview times from over 60 minutes to around 20 minutes. No new interviews or observations were added to the process after the third site visit, indicating the we had reached saturation for the interview guide. In total across the five medical schools we interviewed 83 individuals involved in all aspects of doctoral programmes, reviewed 40 documents, and visited laboratories, libraries, computer centres, and field sites.

The final evaluation process consisted of interviews using the developed guide (i.e., a grid with questions mapped against specific interviewees) (see Text S1), and a review of documents (e.g., policies, regulations, course handbooks) and facilities (e.g., laboratories, libraries, computer centres) to corroborate information from the interviews. The list of interviewees comprised university policy makers (e.g., principals, provosts, deans), researchers (e.g., doctoral students, post-doctoral scientists, research supervisors, research centre staff), and support staff (e.g., ethicists, administrators, accountants, librarians, laboratory scientists). The questions in the final interview guide were compared to the initial list of doctoral programme components we had extracted from the literature to ensure that the iterative adaptations of the process had not resulted in any major omissions.

## Outcomes of the Evaluation Process

At the end of the site visits the doctoral programme coordinator at each university was provided with a confidential report about their own institution and also an anonymised overview report that amalgamated and summarised the key findings from the individual institutional reports. The institutional reports provided a narrative account of corroborated interviewee responses, a list of gaps in doctoral programme provision, and potential remedies proposed by the interviewees. It was possible to identify gaps in provision because our evaluation process was based on an extensive literature review and was therefore a “benchmark” that contained all the elements needed to run a successful doctoral programme.

Following the evaluation process the institutions used the evaluations to develop and implement their own plans to remedy the gaps in capacity and to derive indicators that could be used to monitor progress. Evidence suggests that these indicators will need to be revised regularly as the universities' doctoral programmes mature and become more sophisticated [8]. The capacity gaps that occurred in more than one institution, and potential remedies to address these gaps, have been amalgamated in Table 1. Interestingly, over half of these gaps could potentially be addressed by the universities themselves without any additional external resources (e.g., see Box 3). To address other capacity gaps, such as lack of access to suitable electronic resources and the inexperience of many of the supervisors, additional external inputs would be required (e.g., see Box 4). Using the recommendations in the report, each university coordinator prioritised the areas identified as needing support, identified the steps that would be needed to fill these gaps, and estimated the costs of doing so. The MCDC programme will be able to finance some of these action plans. By applying this standardised evaluation process to several institutions it was also possible to identify common capacity gaps (Box 5), which could become the focus of cross-institutional efforts by external agencies.

**Box 3. Case Study 1**

The doctoral programme evaluation was conducted in an African university that had just started to run its own doctoral programmes. The evaluation revealed that information for students about what was expected of them, how the programme was organised, and what resources were available to them was lacking. Some documents were available in the university, but they were incomplete, not specifically for students in the medical college, and difficult for students to access. The programme coordinator reviewed doctoral students' handbooks from several sources and also used our doctoral programme evaluation interview guide to develop a comprehensive student handbook for doctoral students, which was made available on their intranet.

**Box 4. Case Study 2**

One of the universities had a particular problem with slow and unreliable Internet access. Although this had long been recognised as a problem in the institution, the doctoral evaluation process revealed that every cadre of interviewee mentioned it as a major problem, not only for research students but also for undergraduates and staff. This weight of evidence collected by an external team enabled the university to make a case for several funders to join forces to provide a fast broadband connection for the university.

**Box 5. Common Gaps in Capacity for Doctoral Programmes in African Universities**

- Incomplete supporting documentation such as policies, regulations, and handbooks; documents sometimes inappropriately combined with handbooks for Master's courses and often not easily available to students and/or staff
- Lack of suitably qualified academic faculty with experience of supervising doctoral students
- Inadequate resources (such as books, journals, computers, and Internet access) to support doctoral programmes; staff responsible for providing these resources were generally not involved in planning for the doctoral programme, so they were not able to cater to the needs of these students
- Lack of dedicated desk space for doctoral students and very little formal opportunity for mutual support
- Lack of a formal induction programme to make students aware of, for example, the requirements of the programme or the availability of resources to support their studies
- Lack of a systematic skills development programme within the institution for either supervisors or students
- Unclear mechanisms for identifying and managing students who were failing to progress or who had missed their completion dates
- Excessive time taken to complete the final examination process (often exceeded 12 months)
- Students being unaware of appeal processes that were independent of their supervisor(s)
- Lack of mechanisms for soliciting feedback from students and staff or for using this routinely to enhance the programme

**Table 1:** Potential remedies for gaps that occurred commonly in five African universities' capacity for managing doctoral programmes.

Remedies for Gaps in Universities' Doctoral Programmes	Responsibility for Action	Potential Indicators	Comments
**Institutional arrangements**			
The universities' policies and regulations governing doctoral programmes should cover all aspects of the programme; individual faculties should have their own regulations, based on those of their university, which provide detailed information about all aspects of the programme; each faculty should have a handbook to inform students and staff about all aspects of the programme; these handbooks should be specific to the doctoral programme and should be easily accessible by students and supervisors	African university	Comprehensive policies and regulations available; comprehensive course handbook available	No external resources needed
Non-academic staff who support post-graduate programmes such as information technology, library, and administration staff, should be involved in planning services for post-graduate programmes; the skills and numbers of these staff, as well as their facilities, need to expand in parallel with the post-graduate programmes	African university	Cross-disciplinary committee established; minutes of meetings available	External resources may be needed to develop skills and facilities
**Research environment**			
Arrangements should be made for students to have access to an appropriate range of electronic resources through partner institutions in high-income countries or other alternative mechanisms, until local systems are adequate	MCDC and other funders	Positive feedback on student satisfaction survey	Additional funds may be required
Dedicated learning space with Internet connectivity should be made available to doctoral students, and a regular programme of academic discussions between students and faculty instigated	African university	Suitable space available; regular PhD student meetings	No/few external resources needed
**Selection/admissions**			
A formal induction should be provided for doctoral students	African university	Evidence of induction course	No external resources needed
Adequate funding should be available for the whole duration of the student's doctoral programme	African university	Budgets; financial statements	University resources in collaboration with PhD funders
**Supervision**			
A regular programme of workshops on supervisory skills combined with opportunities for peer support and mentoring should be provided to faculty supervising doctoral research; the roles and responsibilities of joint supervisors should be agreed and documented	MCDC and other funders	Evidence of supervisor training	Significant planning and coordination needed to develop and deliver course
A database of PhD supervisors in African institutions and their specialist skills should be created to expand the pool of available supervisors in the region	MCDC and other funders	Database available	Resources needed to set up database and to fund visits between African institutions
Faculties should actively promote engagement of policy makers and research users in determining research topics and in utilising research results	African university	Priority research areas agreed on and disseminated	External agencies can also be advocates
**Skills development**			
A formal skills development course should be provided that systematically covers the areas needed by doctoral students including “writing for publication”; consideration should be given to awarding credits for this course	African university	Evidence of skills training	Significant planning and coordination needed to develop and deliver course
**Assessment**			
Progress and completion rates for students should be monitored closely, and the causes for non-progression should be thoroughly investigated and addressed	African university	Progress database available	Addressing causes of non-progression could be included in institutions' annual plans
**Student representation, welfare, and appeals**			
Avenues should be created for student concerns to be addressed by individuals who are independent of their supervisors	African university	Information included in handbook	No external resources needed
Provision should be made for students with disabilities	African university	Information included in handbook	Likely to require additional resources
**Feedback and evaluation**			
A process should be put in place for regular review and enhancement of the PhD programme	African university	Minutes of annual review meeting	No external resources needed
Plans and targets for developing institutional research capacity should be developed and used to regularly monitor progress	African university	Minutes of annual review meeting	No external resources needed

## Limitations of the Evaluation Process

The quantitative indicators we identified for monitoring progress in strengthening institutional capacity (e.g., number of students; time to complete course; number of publications, grants, or presentations) can be measured relatively easily, but they do not adequately capture factors that contribute to developing an enabling environment for research [9]. Our evaluation process therefore also included some qualitative indicators (e.g., student satisfaction, quality of learning spaces), although we recognise that these may be more difficult to measure than quantitative indicators. This strategy of combining qualitative and quantitative indicators is similar to the approach taken in the few other published studies that have evaluated research capacity development [7].

The evaluation process was developed with and for health faculties in universities in developing African countries, and it has not been evaluated beyond this context. Nevertheless, because the key components outlined in the overview (Figure 1) were derived from the global literature, the process is likely to be applicable to doctoral programmes in faculties and universities outside Africa. However, the specific types of capacity gaps may vary between countries with different levels of socio-economic development (e.g., slow Internet access and lack of doctoral research supervisors were common gaps in our five African institutions, but this may not be the case in other regions).

## Conclusion

We have developed a comprehensive evidence-based process for evaluating all the policies and systems required for doctoral programmes. Unlike previously published methods for evaluating doctoral programmes, our process incorporated the perspectives of students, staff, the local research community, and the universities' policy makers and was applicable across different countries and programmes of differing maturity. Our standardised evaluation process not only enabled the universities to develop and monitor strategies to address their own capacity gaps but also provided them with a mechanism for justifying, planning, commissioning, and monitoring inputs by external funders while retaining leadership of the process. Unlike the traditional, externally controlled accountability imposed by international donors' agendas, there is evidence that this “endogenous accountability” type of monitoring is likely to promote better ownership and performance [10].

## Supporting Information

Text S1 **Interview guide for evaluating universities' capacity to manage doctoral programmes.**
(DOC)

## Abbreviations

MCDC: Malaria Capacity Development Consortium
