# Supplementary material for: Assessing and Strengthening African Universities' Capacity for Doctoral Programmes
Source: PLoS Med. 2011 Sep 13;8(9):e1001068. doi: 10.1371/journal.pmed.1001068 (PMC3172246; doi:10.1371/journal.pmed.1001068)
Supplement: Text S1 — Interview guide for evaluating universities' capacity to manage doctoral programmes. (DOC) [file pmed.1001068.s001.doc]

**Text S1**

Interview guide for evaluating university’s capacity to manage doctoral programmes (grey boxes indicate which questions should be put to different categories of interviewee). Responses to the questions and any additional comments should be summarised in a narrative report. Gaps in the doctoral programme provision and suggestions from interviewees for addressing these gaps (and which have been verified by others) should be incorporated in the report and summarised as a list of recommendations.

|  | **Questions** | **People to be interviewed** | | | | | | | | | |
| --- | --- | --- | --- | --- | --- | --- | --- | --- | --- | --- | --- |
|  | **INSTUTIONAL ARRANGEMENTS** | Principal  /Provost | PG Dean | Ethics Committee | Administration/ Finance | IT unit | Library | Laboratory manager | External research  units/ affiliates | Supervisors | PhD students |
| 1.1 | Is there a list of institutional policies, regulations and codes of practice for research programmes?* Is it available to the academic staff? And the students? Are the staff/students aware of these documents? |  |  |  |  |  |  |  |  |  |  |
| 1.2 | How many doctoral students do you plan to recruit?  How many will have institutional/in-country funding? How many will have external funding? |  |  |  |  |  |  |  |  |  |  |
| 1.3 | Are student stipends available? What are the conditions on these? |  |  |  |  |  |  |  |  |  |  |
| 1.4 | Is there monitoring for completion rates within a specified time frame?*  Are penalties clearly stated? |  |  |  |  |  |  |  |  |  |  |
| 1.5 | Is there a nominated person responsible for overseeing doctoral programmes? Do they have terms of reference?* Do departments have a Director of PG research and/or a Departmental Research Committee?* What is their relationship to each other? |  |  |  |  |  |  |  |  |  |  |
| 1.6 | Does the university have any affiliated research institutions? |  |  |  |  |  |  |  |  |  |  |
|  | **RESEARCH ENVIRONMENT** | Principal  /Provost | PG Dean | Ethics Committee | Administration/ Finance | IT unit | Library | Laboratory manager | External research  units/ affiliates | Supervisors | PhD students |
| 2.1 | Can you offer students…?*   - Library support - Access to electronic journals and up to date text books - Laboratory space and equipment - Internet access and computer availability - Learning about on-line searches - Student support services (e.g. counselling, health services, academic skills support, disability support |  |  |  |  |  |  |  |  |  |  |
| 2.2 | Is there an active research environment and what is the evidence?* |  |  |  |  |  |  |  |  |  |  |
| 2.3 | What support is there for students for biostatistics and database development? |  |  |  |  |  |  |  |  |  |  |
| 2.4 | How are consumables (e.g. laboratory supplies) funded? |  |  |  |  |  |  |  |  |  |  |
| 2.5 | What is the structure and terms of reference for the ethics committee? What is the ethics approval /application process? What is the turn-around time? |  |  |  |  |  |  |  |  |  |  |
| 2.6 | Is the library able to support doctoral students? Are there plans to develop the library? |  |  |  |  |  |  |  |  |  |  |
| 2.7 | Is there dedicated study space for doctoral students?* |  |  |  |  |  |  |  |  |  |  |
|  | **SELECTION/ ADMISSIONS** | Principal  /Provost | PG Dean | Ethics Committee | Administration/ Finance | IT unit | Library | Laboratory manager | External research  units/ affiliates | Supervisors | PhD students |
| 3.1 | How do you recruit doctoral students?* What are the selection and admission policies?* Are they available? How is adherence monitored? |  |  |  |  |  |  |  |  |  |  |
| 3.2 | How are staff trained to do student selection? What are the interviewing procedures?*  What is the timescale for admissions decisions? |  |  |  |  |  |  |  |  |  |  |
| 3.3 | How are opportunities for bursaries communicated to students? How is this funding allocated to students? |  |  |  |  |  |  |  |  |  |  |
| 3.4 | Is there a doctoral handbook for staff?* What does it cover? Is there a doctoral handbook for students?* What does it cover? |  |  |  |  |  |  |  |  |  |  |
| 3.5 | What is the formal induction process for new students?  What does it cover? |  |  |  |  |  |  |  |  |  |  |
| 3.6 | Can students do a part-time PhD? Does this affect funding? |  |  |  |  |  |  |  |  |  |  |
|  | **SUPERVISION** | Principal  /Provost | PG Dean | Ethics Committee | Administration/ Finance | IT unit | Library | Laboratory manager | External research  units/ affiliates | Supervisors | PhD students |
| 4.1 | How are research topics chosen? Are they aligned to national priorities? |  |  |  |  |  |  |  |  |  |  |
| 4.2 | How are supervisors appointed? What policies exist to guide this process?* |  |  |  |  |  |  |  |  |  |  |
| 4.3 | What is the experience (e.g. publication history, research grants) of supervisors? |  |  |  |  |  |  |  |  |  |  |
| 4.4 | How many formal supervisors does each student have? Does the institution have guidelines for joint supervision?* Is there a clear process for resolving any possible conflicts between supervisors? |  |  |  |  |  |  |  |  |  |  |
| 4.5 | What training is given to new supervisors? Is it compulsory? Is there mentoring for supervisors? |  |  |  |  |  |  |  |  |  |  |
| 4.6 | Are there guidelines that exist for students and supervisors about the way that supervision works?* Do they explain the student’s responsibilities? |  |  |  |  |  |  |  |  |  |  |
| 4.7 | What are the mechanisms for monitoring and supporting student progress, including formal review stages? |  |  |  |  |  |  |  |  |  |  |
| 4.8 | What are the procedures for managing students who do not perform to the expected standard?* Who is responsible for identifying and dealing with problems of progression? |  |  |  |  |  |  |  |  |  |  |
| 4.9 | Is there a process in place for students to change their supervisor? |  |  |  |  |  |  |  |  |  |  |
|  | **SKILLS DEVELOPMENT** | Principal  /Provost | PG Dean | Ethics Committee | Administration/ Finance | IT unit | Library | Laboratory manager | External research  units/ affiliates | Supervisors | PhD students |
| 5.1 | How do students identify and review development needs, record personal progress and reflect on application of the skills they have acquired (i.e. personal development planning)? |  |  |  |  |  |  |  |  |  |  |
| 5.2 | What opportunities are provided for doctoral students for personal and professional development? Is any of this training compulsory? |  |  |  |  |  |  |  |  |  |  |
|  | **ASSESSMENT** | Principal  /Provost | PG Dean | Ethics Committee | Administration/ Finance | IT unit | Library | Laboratory manager | External research  units/ affiliates | Supervisors | PhD students |
| 6.1 | What are the procedures for converting MPhil to PhD (if appropriate)?* |  |  |  |  |  |  |  |  |  |  |
| 6.2 | How are students made aware of the assessment criteria used to mark dissertations? Are they easily available? Is the hand in date specified? |  |  |  |  |  |  |  |  |  |  |
| 6.3 | Are the assessment criteria clear, fair and consistently applied? Are they appropriate for the level of work? |  |  |  |  |  |  |  |  |  |  |
| 6.4 | Are there clear statements about academic honesty, collusion and plagiarism?* What are the penalties for not adhering to these policies? How are student made aware of the penalties? |  |  |  |  |  |  |  |  |  |  |
| 6.5 | What is the expected turn-around time for feedback on thesis drafts? |  |  |  |  |  |  |  |  |  |  |
| 6.6 | How are external examiners appointed? Is there a transparent moderation process? What is the stated time frame between handing in the completed thesis, and the viva/ result? What are the arrangements for the viva or defence of the thesis, if required? Who is responsible for making the arrangements? |  |  |  |  |  |  |  |  |  |  |
|  | **STUDENT REPRESENTATION AND WELFARE** | Principal  /Provost | PG Dean | Ethics Committee | Administration/ Finance | IT unit | Library | Laboratory manager | External research  units/ affiliates | Supervisors | PhD students |
| 7.1 | What are the procedures for doctoral student representation?* How are student concerns managed and resolved? |  |  |  |  |  |  |  |  |  |  |
| 7.2 | What are the procedures for dealing with student complaints? How students are made aware of these procedures? Are students able to complain without fear of being penalised in any way? |  |  |  |  |  |  |  |  |  |  |
| 7.3 | Does each doctoral student have an independent advisory panel? What are the terms of reference and the composition of the panel? |  |  |  |  |  |  |  |  |  |  |
| 7.4 | Is there access to the course and support for students with disabilities? |  |  |  |  |  |  |  |  |  |  |
|  | **APPEALS** | Principal  /Provost | PG Dean | Ethics Committee | Administration/ Finance | IT unit | Library | Laboratory manager | External research  units/ affiliates | Supervisors | PhD students |
| 8.1 | What is the appeals process for doctoral students? How do students know about this process, and when it is appropriate to use it? |  |  |  |  |  |  |  |  |  |  |
| 8.2 | Is there an independent body outside the university to whom the student may have access, if it is required? |  |  |  |  |  |  |  |  |  |  |
|  | **FEEDBACK AND EVALUATION** | Principal  /Provost | PG Dean | Ethics Committee | Administration/ Finance | IT unit | Library | Laboratory manager | External research  units/ affiliates | Supervisors | PhD students |
| 9.1 | Is there a formal process fro evaluating the doctoral programme? Whose responsibility is it to ensure that evaluations take place? |  |  |  |  |  |  |  |  |  |  |
| 9.2 | What opportunities are there for people to provide feedback? Is feedback actively sought and from whom? |  |  |  |  |  |  |  |  |  |  |
| 9.3 | How are the results of this feedback analysed and disseminated? |  |  |  |  |  |  |  |  |  |  |
| 9.4 | How are these evaluations used to make changes to the doctoral programme? Is there a formal process for this to happen? |  |  |  |  |  |  |  |  |  |  |

* verify by reviewing relevant documents or observing facilities such as library, computer laboratory and research laboratories including those of any external organisations.
